# A simple technique to reinforce kinked South Pole RAE tracheal tube

**DOI:** 10.1007/s10877-020-00525-8

**Published:** 2020-05-15

**Authors:** Neeraj Kumar, Prakash K. Dubey, Neha Nupoor, Abhyuday Kumar, Ansharul Haq

**Affiliations:** 1 All India Institute of Medical Sciences, Patna, Bihar India; 2 Indira Gandhi Institute of Medical Sciences, Patna, Bihar India

To the editor,

A preformed South Pole oral Ring–Adair–Elwyn (RAE) tracheal tubes is used in orofacial surgery to provide an unobstructed view of the surgical field. The distinguishing feature of RAE tubes in comparison to standard tracheal tubes is their pre-formed bend. The pre-forming during manufacturing reduces the risk of kinking and obstruction. A black marker bar is imprinted on the RAE tracheal tube at the point of maximum angle of the bend.

We describe a technique to prevent kinking of preformed south polar uncuffed soft seal tracheal tube. A 24 months-old 10-kg body-weight, female child was posted for cleft palate surgery under general anesthesia. After institution of routine monitoring, a 4.0 mm ID preformed south polar tracheal tube (Portex®; Smith Medicals International limited, Hythe, Kent, UK) was used for securing the airway. A Dingman mouth gag retractor was applied to facilitate surgical exposure. About 30 min after start of the surgery we noticed increase in airway pressure from 13 cmH_2_O to 29 cmH_2_O. A quick examination revealed that the south polar tracheal tube was kinked at the point of maximum bend due to compression from Dingman retractor. On removing the retractor airway pressure returned to the previous readings. However, it reappeared after a few minutes at the same point after applying the retractor again.

**Fig. 1:**
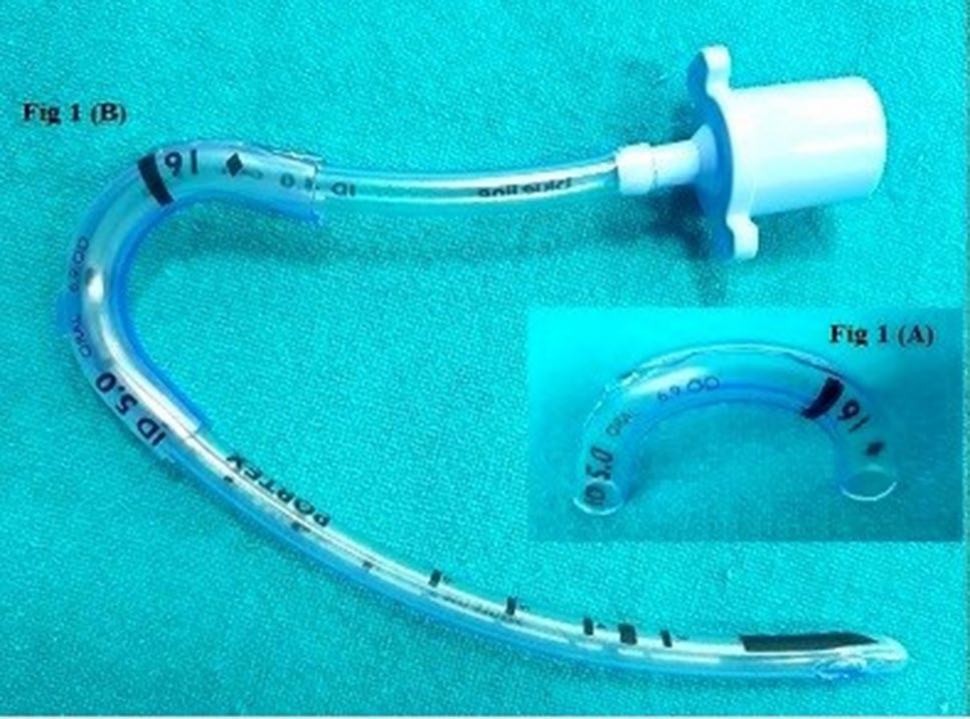
**a** Split piece of another south polar RAE tracheal tube. **b** Kink in the South Polar RAE tracheal tube was corrected by using the split piece of another similar tracheal tube as an external reinforcement

To correct this problem, we cut a 3.5 cm length of another south polar RAE tracheal tube with an ID of 5.0 mm. A cut was made in this piece along the long axis opposite to the radio-opaque marker (Fig. 1a). This piece was now spread apart and gently slipped over the kinked segment of the RAE tracheal tube in situ, keeping the radioopaque mark on the side of the kink (Fig. 1b). This worked as a reinforcement over the kinked part. Thereafter, the course of anesthesia and recovery were uneventful.

Anesthesiologists must be aware of the possibility of airway obstruction that might occur with the use of Dingmans mouth gag retractor. These retractor are self-retaining retractor that includes two cheek retractors and a tongue blade of various sizes as attachment. In this child size 1 tongue blade was used. This tongue blade also contains a groove for the lodgement of the tracheal tube.

Possible contributing factors that may result in this unusual problem are structural defect in the tube at the point of maximum bend, faulty technique of application of the retractor, and operation room temperature. If the TT’s temperature increases to 36 °C, the tube softens making it liable to kinking [1]. The esophageal temperature recorded in this case was 38.5 °C. We suspect that the tube got softened at this temperature that contributed to rekinking even after repositioning of the retractor.

The technique by Dubey and Kumar [2] is simple and quick to perform with the help of easily available resources in the operating room. A larger size of tube was selected so that it snugly fits on the outer circumference of the kinked tube in situ. We have also observed that kinking of thermally softened tubes occur more on bending it in the direction of the convexity of the tube than the concavity.

Key advantages of using another RAE tracheal tube as reinforcement:

1. It provides additional cushion at point of maximum bending without any kinking or tracheal tube narrowing.
2. This pre-cut south polar RAE tracheal tube splint can be used in presence of warming devices.
3. Use of this reinforcement can avoid the need for replacing the kinked tube intraoperatively.
